# Outcomes of Corneal Compound Myopic Astigmatism with Presbyopia by Zeiss PRESBYOND^®^ Laser Blended Vision LASIK Using Default CRS-Master^®^ Target Refractions for Reduced Anisometropia

**DOI:** 10.3390/jcm13103011

**Published:** 2024-05-20

**Authors:** Julia Hernández-Lucena, Federico Alonso-Aliste, Jonatan Amián-Cordero, José-María Sánchez-González

**Affiliations:** 1 Department of Physics of Condensed Matter, Optics Area, University of Seville, Reina Mercedes S/N, 41012 Seville, Spain; jsanchez80@us.es; 2 Department of Ophthalmology (Tecnolaser Clinic Vision^®^), Refractive Surgery Center, Juan Antonio Cavestany, 41018 Seville, Spain; federicornea@gmail.com (F.A.-A.); dramian@tecnolasersevilla.es (J.A.-C.)

**Keywords:** PRESBYOND^®^ Laser Blended Vision, myopic presbyopes, micro-anisometropia, presbyopia treatments, uncorrected distance visual acuity (UDVA), corrected distance visual acuity (CDVA), binocular summation

## Abstract

**Background/Objectives:** Presbyopia, a common age-related refractive error, affects over a billion people globally and significantly impacts daily life. **Methods:** This retrospective study analyzed 288 eyes of 144 patients undergoing LBV PRESBYOND^®^ treatment for myopic presbyopia with astigmatism, aiming to evaluate precision, efficacy, safety, and stability over six months. **Results:** Key findings include high efficacy, with 99% of distance-eyes achieving uncorrected distance visual acuity (UDVA) of 20/25 or better, and 85% of near-eyes achieving UDVA of 20/32 or better. The results show excellent refractive outcomes, with 99% of long-sighted eyes and 97% of near-sighted eyes having a postoperative spherical equivalent within ±1.00 D. Safety was demonstrated by no loss of two or more Snellen lines after treatment, with 94% of patients maintaining corrected distance visual acuity (CDVA) before and after surgery. **Conclusions:** Overall, LBV PRESBYOND^®^ proved effective, safe, and well tolerated for myopic presbyopia correction, offering satisfactory visual outcomes and potential spectacle independence for various distances. This study underscores the importance of individualized treatment based on patient age, highlighting the positive impact of binocular summation on visual function. This study contributes to the growing body of evidence supporting LBV PRESBYOND^®^ as a viable option for addressing presbyopic myopia, offering insights into its efficacy and safety profile. Further research could explore postoperative stereopsis and long-term outcomes to enhance understanding and refine treatment protocols.

## 1. Introduction

Presbyopia is a refractive error that arises due to the aging of the eye, resulting in the loss of the ability to focus on nearby objects [1]. Numerous strategies exist for correcting presbyopia, encompassing options such as corrective glasses [2], contact lenses [3], pharmacological treatment [4], or corneal or intraocular surgery [5]. Presbyopia appears after the age of 40 and is the main cause of vision loss around the world, with more than one billion people affected, approximately a quarter of the world’s population [6]. A systematic review conducted in 2015 explored its prevalence and impact, revealing that 826 million individuals with presbyopia experienced poor near vision due to uncorrected or inadequately corrected distance vision [6]. This deficiency translates into significant disability for both affected individuals and society at large [7].

The use of refractive surgery as a treatment option has been a subject of considerable discussion within the field of ophthalmology in recent years. Laser Blended Vision (LBV) with PRESBYOND^®^, a micro-anisometropia refractive surgery technique, stands out as a prominent method based on a non-linear aspheric ablation profile developed by Carl Zeiss Meditec [8]. This approach, available on the Mel 80 and Mel 90 platforms, is widely acknowledged for its effectiveness and safety [9]. What sets PRESBYOND^®^ apart from other techniques is its foundation upon the creation of micro-anisometropia coupled with an improvement in depth of focus through the modification of spherical aberration [10]. The increase in spherical aberration is maintained within acceptable thresholds to ensure the preservation of satisfactory distance visual acuity (VA) and contrast sensitivity [11]. The primary objective of this technique is to reach emmetropia in the dominant eye, while targeting a refraction of −1.50 diopters (D) for the non-dominant eye [12]. The Custom Refractive Surgery Master (CRSM) is a set of guidelines and protocols aimed at guiding the practice of refractive surgery tailored to the individual needs of each patient. This approach focuses on minimizing any residual refractive error after surgery, considering factors such as the patient’s age. [13]. It is imperative to underline that this treatment yields a blended vision zone, involving both near and distant visual fields, thereby helping favorable outcomes in intermediate vision [14]. For a comprehensive assessment of visual function, it is essential to incorporate evaluations incorporating distance and near visual acuity, as well as assessments of motor and sensory dominance and stereopsis [15].

The aim of our retrospective study was to analyze and assess the accuracy, effectiveness, safety, and stability of laser refractive treatment for presbyopic myopic patients over a six-month follow-up period.

## 2. Materials and Methods

### 2.1. Study Design

This is a single-arm, retrospective, longitudinal clinical study. All patients included in this study were informed verbally and in writing about the benefits, characteristics, and risks of the surgeries and signed an informed consent form prior to surgery. This study complies with the standards of the Declaration of Helsinki and has the favorable report of the Research Ethics Committee of the Virgen Macarena and Virgen del Rocio University Hospitals in Seville, Spain, with promoter code 0863-N-22.

### 2.2. Subjects

Presbyopic myopic patients with or without astigmatism submitted to the Zeiss PRESBYOND^®^ LBV technique based on CRSM recommendations from January 2016 to December 2021 were retrospectively recruited. All surgeries were performed at the Tecnolaser Clinic Vision Ophthalmology Center in Seville, Spain. The follow-up period for these patients was six months.

Inclusion criteria for the study group were patients of either sex, aged 40−60 at the time of surgery; presbyopia with myopia, with or without astigmatism; patients with a subjective sphere refraction equal to or less than −7.00 D of myopia and/or −5.75 D of astigmatism; corrected distance visual acuity (CDVA) of both eyes equal to or greater than 0.10 logarithm of the minimum angle of resolution (LogMAR); normative stereopsis, equal to or less than 400 arc seconds; and patients with keratometry greater than 38 D and under 48 D. Subjects excluded from the study were patients who had previously undergone any other refractive surgery.

### 2.3. Preoperative Assessments

Before surgery, a complete optometric and ophthalmological study was performed on all patients. In the optometric study, subjective refraction distance and near cycloplegic refraction were measured with an autorefractometer (KR8800, Topcon, Tokyo, Japan); motor and sensory dominance were also measured, alongside stereopsis with the TITMUS test (2007, Vision Assessment Corporation, Elk Grove Village, IL 60007, Estados Unidos, USA). A cover and uncover test was carried out with a translucent occluder (Optometric Promotion, Burgos, Spain) and near point of convergence was measured. Other tests were topography and tomography measured with a Pentacam AXL^®^ (Oculus, Wetzlar, Germany), intraocular pressure and corneal biomechanics with Corvis ST^®^ (Oculus, Wetzlar, Germany), and epithelial thickness and retina measured with Optovue optical coherence tomography (OCT) (OftalTech, Barcelona, Spain). Ophthalmological examination included evaluation with a slit lamp biomicroscope assessment LH-2000 (Indo, Barcelona, Spain) and fundoscopy (Keeler SL4 4AA, Windsor SL4 4AA, Reino Unido, UK) with cycloplegic dilation.

### 2.4. Eye Dominance Test

This test was performed using two methods, one for sensory dominance and one for motor dominance. Sensory dominance was measured with the blur test by adding a positive lens of +1.00 to the subjective refraction of the patient, firstly in one eye and then in the other. The eye that perceives the most blurring will be the sensory dominant eye [16,17]. To measure motor dominance, we use the hole-in-card test, which involves the patient extending both arms to hold a card with a hole in the center. The patient then looks at a letter on an optotype through the hole with both eyes open. We cover one eye and then the other to determine which eye maintains focus on the optotype. The eye that continues to see the letter on the optotype will be the motor dominant eye [18,19].

### 2.5. Micro-Anisometropia Evaluation

The attempted refraction for each patient after surgery was emmetropic for the dominant eye and a negative residual, determined by a micro-anisometropia tolerance test and considering the patient´s age, in their non-dominant eye. In the preoperative examination, tolerance to micro-anisometropia was measured with test glasses so that the surgeon could correct the maximum amount tolerable by the patient that allowed good uncorrected near visual acuity (UNVA) without losing uncorrected distance visual acuity (UDVA) [20].

The patient’s maximum tolerance was initially tested using a +1.50 D lens in the non-dominant eye. If the patient did not experience distance blurring, we incrementally added positive lenses in 0.25 steps until minimal blur was noticed. Conversely, if distance blurring occurred with the +1.50 D lens, we incrementally introduced negative lenses in −0.25 steps until the patient achieved comfortable distance vision. Patients who did not tolerate +1.00 D or less with test glasses performed monovision tests with Biofinity-brand contact lenses (Cooper Vision, Pleasanton, California) for three to four days. Patients who did not tolerate micro-anisometropia tests with +1.00 D contact lenses were not suitable to be intervened on with the Zeiss PRESBYOND^®^ LBV technique based on CRSM.

### 2.6. Procedure

The patients were informed that they would undergo the same treatment as those included in a previous study performed by our research team [21] and the same postoperative protocol was followed. Those patients who did not finish adjusting to near and distance vision at the three-month visit were ordered to carry out accommodation/integration exercises [22]. Hart Letters were sent to those who found it difficult to focus when changing their vision from far to near or vice versa to work on accommodation. Red/green glasses with the reading bars were provided to those who struggled to integrate images at both far distance and near-distance, aiming to enhance fusion.

### 2.7. Surgical Technique

All surgical procedures were performed by two surgeons with experience in presbyopia laser correction (FA-A, JA-C) using the VisuMax^®^ (Carl Zeiss Meditec AG, Jena, Germany, 2010) femtosecond laser and the Mel^®^ 90 (Carl Zeiss Meditec AG, Jena, Germany, 2014) excimer laser.

Planning was conducted in the Custom Refractive Software (CRS) Master (Zeiss, Jena, Germany) of LBV PRESBYOND^®^, a system that calculates a personalized pattern of laser treatment considering the patient’s refractive correction, functional age, corneal shape and thickness, pupil size, and optical aberrations. It is a binocular treatment that combines aberrometry and topography. The data obtained by aberrometry are transferred electronically to the excimer laser to perform the appropriate ablation for each patient. The guided wavefront treatment consists of leaving the dominant eye emmetropic with or without implementation of spherical aberrations. In the non-dominant eye, an determination of spherical aberration, residual defocus considering age, keratometry, lens densitometry, ametropia, stereopsis, pupil, initial spherical aberration, and prognosis is performed. The PRESBYOND^®^ technique stands apart from other refractive surgeries by specifically targeting the induction of controlled spherical aberrations. These aberrations are intentionally introduced to enhance the eye’s depth of focus, resulting in clearer vision at both near and far distances. By broadening the depth of focus, PRESBYOND^®^ spherical ablation aims to deliver sharper vision and improved focusing capabilities across various distances. Typically performed as an outpatient procedure, it involves the use of topical anesthesia to minimize patient discomfort. Following surgery, patients can anticipate a significant enhancement in their vision for both near and distant objects, potentially reducing or even eliminating the need for corrective eyewear like glasses or contact lenses. It is important to recognize that the success of the procedure and visual outcomes may vary based on individual patient characteristics and the surgeon’s expertise. Prior to undergoing any surgical intervention, patients should undergo a thorough evaluation by an ophthalmologist to assess their suitability for refractive surgery and discuss potential risks and benefits.

In this study, a modification of the PRESBYOND^®^ LBV protocol with induced micro-anisometropia according to the CRSM recommendation based on age was performed. With this modification, the treatment aims to create micro-anisometropia based on the patient´s age so that the induced micro-anisometropia ranges between values of −0.75 D and −1.12 D. The goal of this modification of the PRESBYOND^®^ LBV protocol is to decrease the induced micro-anisometropia in the non-dominant eye. A flap was created using the VisuMax femtosecond laser system (Zeiss, Jena, Germany) adjusted to a thickness of 100 microns. 

### 2.8. Statistical Analysis

Statistical analysis was performed with the statistical program SPSS version 29.0 (IBM Corporation, Armonk, NY, USA). Result analysis was performed according to the Standard Charts for Reporting Refractive Surgery. All VA data were changed to Snellen format after calculations were performed in LogMAR format. Student’s test was performed for parametric dependent variables. The results were analyzed separately for data from the dominant eye and data from the non-dominant eye. Refractive stability was assessed by measuring refraction at three and six months and at one year. All statistical tests were performed with 95% confidence levels (*p* < 0.05).

**Figure 1:**
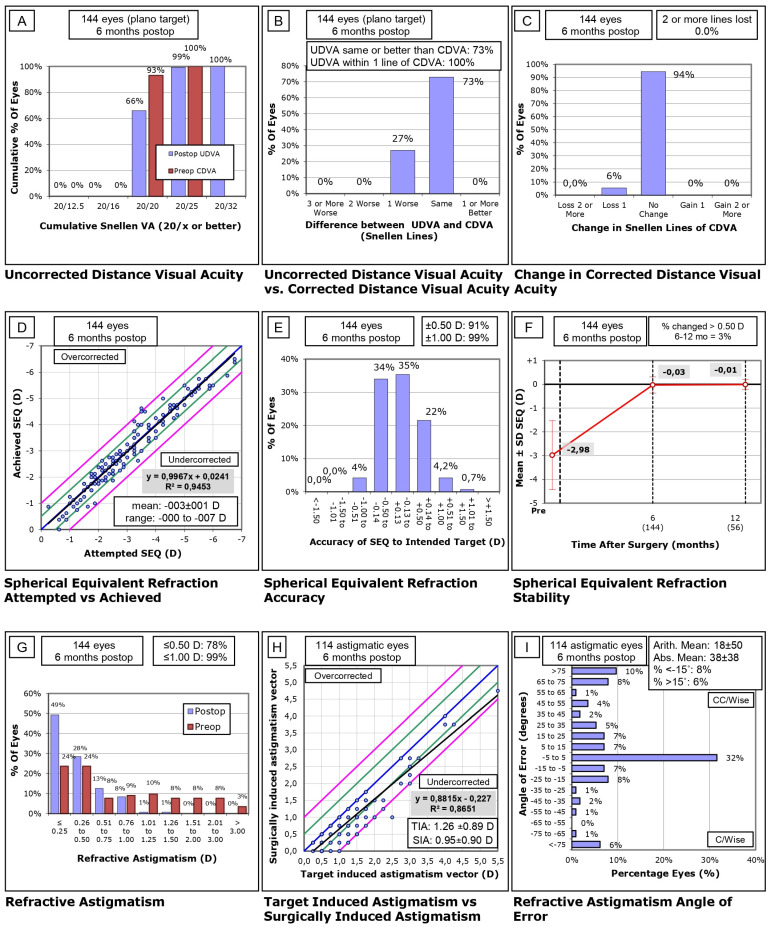
Nine standard graphs of dominant eye for reporting refractive surgery. (**A**) Uncorrected distance visual acuity (UDVA). (**B**) Difference between UDVA versus corrected distance visual acuity (CDVA). (**C**) Change in Snellen Lines of CDVA. (**D**) Spherical equivalent refraction: attempted versus achieved. (**E**) Spherical equivalent refraction accuracy. (**F**) Spherical equivalent refraction stability. (**G**) Refractive astigmatism. (**H**) Target-induced astigmatism (TIA) versus surgically induced astigmatism (SIA) and (**I**) refractive astigmatism angle of error.

**Figure 2:**
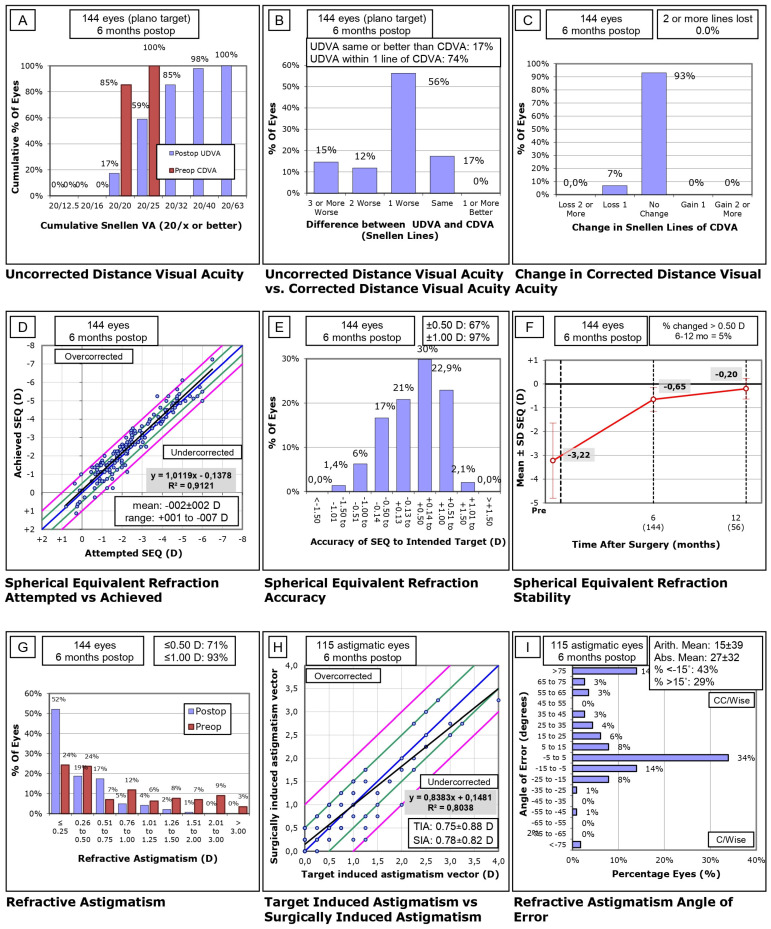
Nine standard graphs of non-dominant eye for reporting refractive surgery. (**A**) Uncorrected distance visual acuity (UDVA). (**B**) Difference between UDVA versus corrected distance visual acuity (CDVA). (**C**) Change in Snellen Lines of CDVA. (**D**) Spherical equivalent refraction: attempted versus achieved. (**E**) Spherical equivalent refraction accuracy. (**F**) Spherical equivalent refraction stability. (**G**) Refractive astigmatism. (**H**) Target-induced astigmatism (TIA) versus surgically induced astigmatism (SIA) and (**I**) refractive astigmatism angle of error.

The results of the refractive surgery were calculated according to the standard graphs originally defined by Waring [23], shown in Figure 1 and Figure 2.

### 2.9. Postoperative Assessments

Refraction was measured with an autorefractometer and confirmed with a subjective monocular, and binocular UDVA and binocular UNVA were checked, with the VA limit being 20/20.

## 3. Results

### 3.1. Demographic and Baseline Characteristics

A total of 288 eyes of 144 patients with a mean age of 47.5 ± 4.05 (40 to 60 years) met the inclusion and exclusion criteria. All the patients completed a six-month follow-up and 38.89% (112 eyes) completed an annual follow-up. Visual and refractive outcomes were studied separated by dominant and non-dominant eye. In the dominant eye, the mean attempt to correct for SE refraction was −2.99 ± 1.45 D (−6.75 to −0.25 D) (*p* < 0.01) and the mean attempted cylinder was −0.98 ± 1.02 D (−5.50 to 0.00 D) (*p* = 0.05). In the non-dominant eye, the mean attempt to correct for SE refraction was −3.22 ± 1.58 (−7.25 to −0.25) (*p* < 0.01) and the mean attempted cylinder was −0.99 ± 0.95 (−4.50 to 0.00) (*p* < 0.01). Descriptive statistics of preoperative and follow-up refraction, keratometry, and pachymetry data are shown in Table 1.

### 3.2. Visual Outcomes

After surgical treatment conducted in distance-eyes, it was shown that 99% of patients achieved UDVA of 20/25 or better (Figure 1A), that 73% of patients achieved identical UDVA after surgery to that before surgery (Figure 1B), and that no subject lost two or more Snellen lines after refractive treatment (Figure 1C). After the treatment was carried out in near-eyes, it was shown that 85% of the patients obtained UDVA of 20/32 or better (Figure 2A), the UDVA was within two lines of the preoperative CDVA in 85% of eyes (Figure 2B), and that 93% of patients had no changes in VA during postoperative follow-up (Figure 2C), considering that refractive objective for near-vision eyes was myopic. Binocularly, all eyes achieved UDVA 20/25 or better and 96.54% achieved 20/20. Binocular UNVA results show that 97.22% of patients achieved VA 20/32 or better.

### 3.3. Refractive Outcomes

Treatment achieved a 99% correction (Figure 1D); the accuracy of the spherical equivalent of 99% of the treated long-sighted eyes was within ±1.00 D and 91% within ±0.50 D (Figure 1E), and 3% of long-sighted eyes had refractive changes greater than 0.50 D at six months after surgery (Figure 1F). A total of 99% of patients had long-sighted-eye astigmatism of ≤1.00 D and 78% of ≤0.50 D (Figure 1G); the treatment achieved an 88% correction (Figure 1H) and in 46% of patients, there was a rotation of 15 degrees or less in the axis of astigmatism of the long-sighted eye (Figure 1I).

Treatment achieved a 100% correction (Figure 2D); in 97% of the near-sighted eyes, a postoperative SE of ±1.00 D was left (Figure 2E), and 5% of the near-sighted eyes had refractive changes greater than 0.50 D six months after surgery (Figure 2F). A total of 93% of patients had astigmatism in near-sighted eyes of ≤1.00 D and 71% of ≤0.50 D (Figure 2G); the treatment achieved an 83% correction (Figure 2H) and 56% of the near-sighted-eye astigmatisms presented an axis rotation of 15 degrees or less (Figure 2I). The mean deviation from the intended SE correction was −0.03 ± 0.35 D (−0.87 to +1.12) in dominant eyes and −0.82 ± 0.08 (−0.75 to −1.12) D in non-dominant eyes.

### 3.4. Safety

The safety of this study is limited by the non-measurement of VA greater than 20/20. All eyes achieved a UDVA of 20/32 or better after refractive treatment and 99% of 20/25 or better; 94% of patients maintained their CDVA before surgery and UDVA six months after surgery, and no subject lost two or more Snellen lines after refractive treatment. Complications were minimal, with superficial keratitis being the most common, occurring in 3.47% of patients, and photophobia reported in 2.78%. The improvement of binocular UDVA compared to monocular UDVA for both distance and near vision at six-month follow-up (Table 1) and at twelve-month follow-up (Table 2) was statistically significant (*p* < 0.01).

## 4. Discussion

**Figure 3:**
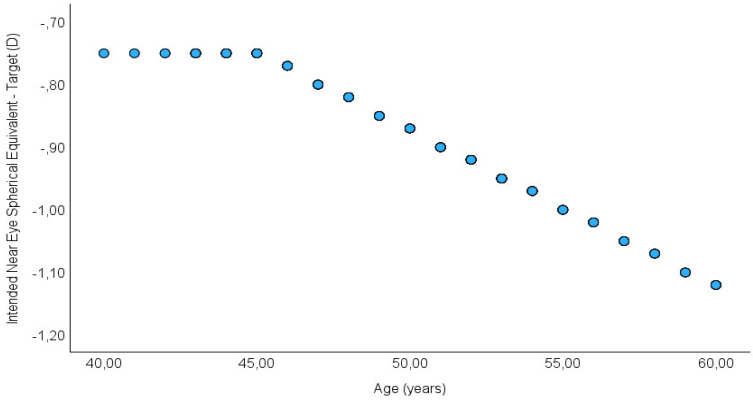
Distribution of intended after-treatment spherical equivalent refraction (target) plotted against age.

Our results demonstrate the efficacy, safety, stability, and precision of LBV refractive treatment using predetermined CRSM target refractions to reduce anisometropia. Additionally, the creation of micro-anisometropia combined with a modification of spherical aberration and an increase in depth of focus is a procedure perfectly tolerated by myopic patients and achieves good UDVA and UNVA. Induced micro-anisometropia is better tolerated by the patient thanks to the induction of spherical aberration, which, by generating an increase in depth of focus, leads to satisfactory visual outcomes in intermediate vision [24,25,26]. To our knowledge, all published studies on PRESBYOND^®^ have shown results after surgery with micro-anisometropia and a myopic target of −1.50, regardless of age [10,13,14,15,27,28,29]. Our study aims to demonstrate that we can decrease micro-anisometropia based on the patient’s age (Figure 3) without worsening visual or refractive outcomes [30].

According to Reinstein et al. [15], a binocular UDVA of 20/20 was achieved in 90% of patients, and J2 near vision was achieved in 89%, while Ganesh et al. [10] reported binocular UDVA of 20/20 in 98% of patients and J2 in binocular UNVA in 83% of patients. Our results were comparable and even better: 96.54% of patients achieved UDVA of 20/20 and 97.22% of patients achieved UNVA of J2 or better. The treatment efficacy was high, and the accuracy of the achieved spherical equivalent compared to the intended target was within ±0.50 D for 91% of long-sighted eyes and within ±1.00 D for 97% of near-sighted eyes.

The results of our study show that the modified protocol of PRESBYOND^®^ LBV with induced micro-anisometropia as per the CRSM recommendation is a well-tolerated and effective treatment for myopic presbyopia; 73% of patients achieved identical UDVA after surgery to their CDVA before surgery, and no subject lost two or more Snellen lines after refractive treatment. The results obtained in non-dominant eyes were as expected, as attempting to leave a myopic residual would result in a decrease in UDVA; however, we interpret these results as optimal, since monocular UDVA is better than UDVA of an eye with the same, untreated refraction. This means that although the induced myopic target in the non-dominant eye causes a loss of UDVA, reaching a UDVA in that eye of 20/63 in some patients, 85% of patients achieved a UDVA of 20/32 or better. Additionally, it is important to highlight that VA measured at six months was better binocularly than monocularly for distance vision, which also demonstrates the retention of certain neural summation despite anisometropia in the eyes [31] and aligns with Althomali et al. [32], a study that complements the findings of Luger et al. [33]: patients experienced significant improvements in UNVA and UDVA with stable results up to three years after surgery. These results coincide with previous studies where the myopic target in the non-dominant eye was higher (−1.50 D) [28]. In the near-vision eye, we achieved one of the goals of the technique, which is to leave a myopic target in 100% of patients with a mean result of −0.82 ± 0.08 (−0.75 to −1.12) D. The lack of need for enhancement in our population suggests good stability of the results obtained, which can be considered a favorable aspect of this procedure, with these results being better than in previous studies by Reinstein et al. [15] or Russo et al. [28]. Comparing our results with other laser treatment techniques for presbyopia, we also demonstrate effective outcomes. PresbyMAX is another laser surgical procedure that corrects presbyopia by modifying the cornea to restore both near and distance vision. Studies on PresbyMAX reported variable outcomes in terms of UDVA and UNVA, with some studies showing lower percentages of patients achieving satisfactory vision compared to LBV, thus demonstrating that LBV suggests superior efficacy and precision in correcting both near and distance vision, as well as better procedural safety [34]. The Supracor technique is an ophthalmic surgical procedure that combines LASIK surgery with corneal modification to correct presbyopia, providing improved vision for both near-distance and far distance. It shows similar results to our study, demonstrating high rates of refractive correction, with a high percentage of patients achieving correction within ±1.00 D of the desired target. Additionally, most patients maintained or improved their visual acuity after treatment, although 14% of patients lost lines of vision [35]. This technique shows good results, but LBV demonstrates better outcomes in terms of visual correction and safety.

Although our study has the limitation of not measuring postoperative stereopsis, Reinstein et al. [36] published results where 68% of patients had postoperative stereopsis of 100 s of arc or better, and 93% had 200 s of arc or better, and Brar et al. [27] obtained similar results: all patients had stereopsis of 140 s of arc or better, and 70% of patients had stereopsis of 60 s of arc or better. Our research team has also explored how preoperative stereopsis influences outcomes in hyperopic patients undergoing PRESBYOND^®^ surgery, finding that its impact on visual results tends to diminish over the long term [37]. Other limitations of this study include incomplete annual follow-up, the absence of VA better than 20/20, the lack of contrast sensitivity measurement, and not performing the validated questionnaire that assesses vision and quality of life. However, the strengths of this study include a large cohort of patients, assessment of dominance, preoperative stereopsis measurements, comprehensive follow-up, and individual analysis of dominant and non-dominant eyes. Future studies could include the analysis of postoperative stereopsis and spherical aberrations, as well as the patient´s vision and quality of life, or analysis of contrast sensitivity after refractive.

## 5. Conclusions

In conclusion, Laser Blended Vision (LBV) with PRESBYOND^®^ using predetermined CRSM target refractions for reduced anisometropia proves to be an effective, safe, and well-tolerated refractive treatment for presbyopic myopic patients. It offers excellent outcomes for UDVA and UNVA with great spectacle independence for all distances. The positive contribution of near vision to distant binocular vision and vice versa highlights the importance of binocular summation, which is fundamental for Laser Blended Vision.

## Figures and Tables

**Table 1:** Descriptive statistics of the preoperative and follow-up refraction data.

Parameter Mean ± SD (Range)	Preoperative	6 Months	*p* Value
Eyes (Patients)	288 (144)	288 (144)	
Distance Eye CDVA (LogMAR)	0.00 ± 0.00 (0.00 to 0.05)		
Near Eye CDVA (LogMAR)	0.01 ± 0.02 (0.00 to 0.05)		
Distance Eye UDVA (LogMAR)	-	0.02 ± 0.33 (0.00 to 0.20)	<0.01
Near Eye UDVA (LogMAR)	-	0.14 ± 0.11 (0.00 to 0.50)	<0.01
Binocular UDVA (LogMAR)	-	0.01 ± 0.08 (0.00 to 0.09)	
Binocular UNVA (LogMAR)	-	0.05 ± 0.89 (0.00 to 0.40)	<0.01
Distance Eye Sphere (D)	−2.50 ± 1.55 (−6.75 to 0.00)	+0.15 ± 0.38 (−0.50 to +1.75)	<0.01
Distance Eye Cylinder (D)	−0.98 ± 1.02 (−5.50 to 0.00)	−0.38 ± 0.35 (−1.50 to 0.00)	*p* = 0.05
Distance Eye Axis (°)	75.21 ± 60.00 (0.00 to 180.00)	59.90 ± 62.83 (0.00 to 180.00)	<0.01
Distance Eye SE (D)	−2.99 ± 1.45 (−6.75 to −0.25)	−0.03 ± 0.35 (−0.88 to +1.12)	<0.01
Distance Eye K_min_ (D)	42.99 ± 1.45 (39.00 to 47.40)	-	<0.01
Distance Eye K_max_ (D)	43.48 ± 1.42 (39.40 to 47.50)	-	<0.01
Distance Eye K_mean_ (D)	43.13 ± 1.41 (39.30 to 47.50)	-	<0.01
Distance Eye CCT (µm)	552.39 ± 30.24 (482 to 626)	-	<0.01
Near Eye Sphere (D)	−2.73 ± 1.65 (−6.75 to +0.50)	−0.45 ± 0.53 (−2.00 to +0.75)	<0.01
Near Eye Cylinder (D)	−0.99 ± 0.95 (−4.50 to 0.00)	−0.41 ± 0.41 (1.75 to 0.00)	<0.01
Near Eye Axis (°)	81.56 ± 64.74 (0.00 to 180.00)	58.89 ± 65.19 (0.00 to 180.00)	*p* = 0.26
Near Eye SE (D)	−3.22 ± 1.58 (−7.25 to −0.25)	−0.65 ± 0.50 (−2.25 to +0.75)	<0.01
Intended target refraction Near Eye (D)		−0.82 ± 0.08 (−0.75 to −1.12)	
Near Eye K_min_ (D)	42.98 ± 1.47 (38.20 to 47.00)	-	<0.01
Near Eye K_max_ (D)	44.03 ± 1.55 (39.50 to 47.80)	-	<0.01
Near Eye K_mean_ (D)	43.50 ± 1.45 (38.90 to 47.40)	-	<0.01
Near Eye CCT (µm)	551.78 ± 30.68 (481 to 627)	-	<0.01

SD: Standard Deviation, CDVA: corrected distance visual acuity, UDVA: uncorrected distance visual acuity, UNVA: uncorrected near visual acuity, LogMAR: logarithm of the minimum angle of resolution, D: diopter, SE: sphere equivalent, K_min_: minimum keratometry, K_max_: maximum keratometry, K_mean_: mean keratometry, CCT: central corneal thickness. *p* value for the comparison between the data at preoperative versus 6 months was calculated.

**Table 2:** Descriptive statistics of the preoperative refraction data and twelve-month follow-up.

Parameter Mean ± SD (Range)	Preoperative	12 Months	*p* Value 12 Months
Eyes (Patients)	288 (144)	112 (56)	
Distance Eye CDVA (LogMAR)	0.00 ± 0.00 (0.00 to 0.05)		
Near Eye CDVA (LogMAR)	0.01 ± 0.02 (0.00 to 0.05)		
Distance Eye UDVA (LogMAR)		21.16 ± 1.72 (0.00 to 0.10)	<0.01
Near Eye UDVA (LogMAR)		29.91 ± 9.74 (0.00 to 0.50)	<0.01
Binocular UNVA (LogMAR)		24.38 ± 6.38 (0.00 to 0.40)	
Refraction, Keratometry, and Pachymetry			
Distance Eye Sphere (D)	−2.50 ± 1.55 (−6.75 to 0.00)	0.15 ± 0.40 (−0.75 to +1.50)	<0.01
Distance Eye Cylinder (D)	−0.98 ± 1.02 (−5.50 to 0.00)	−0.41 ± 0.41 (−1.50 to 0.00)	<0.01
Distance Eye Axis (°)	75.21 ± 60.00 (0.00 to 180.00)	56.86 ± 61.54 (0.00 to 180.00)	0.057
Distance Eye SE (D)	−2.99 ± 1.45 (−6.75 to −0.25)	−0.01 ± 0.21 (−1.13 to +1.38)	<0.01
Distance Eye K_min_ (D)	42.99 ± 1.45 (39.00 to 47.40)		<0.01
Distance Eye K_max_ (D)	43.48 ± 1.42 (39.40 to 47.50)		<0.01
Distance Eye K_mean_ (D)	43.13 ± 1.41 (39.30 to 47.50)		<0.01
Distance Eye CCT (µm)	552.39 ± 30.24 (482 to 626)		<0.01
Near Eye Sphere (D)	−2.73 ± 1.65 (−6.75 to +0.50)	−0.47 ± 0.57 (−1.50 to +0.75)	<0.01
Near Eye Cylinder (D)	−0.99 ± 0.95 (−4.50 to 0.00)	−0.58 ± 0.46 (−1.50 to 0.00)	<0.01
Near Eye Axis (°)	81.56 ± 64.74 (0.00 to 180.00)	70.73 ± 66.60 (0.00 to 180.00)	0.265
Near Eye SE (D)	−3.22 ± 1.58 (−7.25 to −0.25)	−0.21 ± 0.43 (−1.88 to +0.50)	<0.01
Intended target refraction Near Eye (D)		−0.82 ± 0.08 (−0.75 to −1.12)	
Near Eye K_min_ (D)	42.98 ± 1.47 (38.20 to 47.00)		<0.01
Near Eye K_max_ (D)	44.03 ± 1.55 (39.50 to 47.80)		<0.01
Near Eye K_mean_ (D)	43.50 ± 1.45 (38.90 to 47.40)		<0.01
Near Eye CCT (µm)	551.78 ± 30.68 (481 to 627)		<0.01

SD: Standard Deviation, CDVA: corrected distance visual acuity, UDVA: uncorrected distance visual acuity, UNVA: uncorrected near visual acuity, LogMAR: logarithm of the minimum angle of resolution, D: diopter, SE: sphere equivalent, K_min_: minimum keratometry, K_max_: maximum keratometry, K_mean_: mean keratometry, CCT: central corneal thickness. *p* value for the comparison between the data at preoperative versus 12 months was calculated.

## Data Availability

Data available on request due to restrictions. The data presented in this study are available on request from the corresponding author. The data are not publicly available due to copyright issues.
